# Conjoint tobacco and alcohol use and depression among HIV-positive patients in Sedibeng, South Africa

**DOI:** 10.4102/safp.v65i1.5687

**Published:** 2023-05-24

**Authors:** Kenneth E. Akahilem, Olufemi B. Omole

**Affiliations:** 1 Department of Family Medicine, Faculty of Health Sciences, University of the Witwatersrand, Johannesburg, South Africa; 2 Department of Family Medicine and Primary care, Faculty of Health Sciences, University of the Witwatersrand, Johannesburg, South Africa

**Keywords:** conjoint, tobacco, alcohol, depression, HIV

## Abstract

**Background:**

Psychosocial challenges among human immunodeficiency virus (HIV)-positive patients may promote substance use disorders. This study explored the relationship between conjoint tobacco and alcohol use and depression symptoms among HIV positive patients in Sedibeng District, South Africa.

**Methods:**

In a cross-sectional study of 404 participants, a questionnaire collected information on sociodemography, tobacco and alcohol use and depression symptoms. Outcome measures included the prevalence of conjoint tobacco and alcohol use and its association with positive screen for depression.

**Results:**

The mean participant age was 43.2 years. Most completed secondary school (62.9%), were black (99.0%), female (65.8%), unemployed (53.6%) and on antiretroviral therapy (ART) for > 1 year (97.8%). Current tobacco use was reported by 23.3% (*n* = 94) participants with most smoking cigarette (73.7%) and having low nicotine dependence (75.5%). Current alcohol use was reported by 43.6% (*n* = 176) participants, and 36.9% were categorised as harmful users. Only 7.7% (*n* = 31) participants screened positive for depression; the prevalence of conjoint tobacco and alcohol use was 19.6% (*n* = 79) and this was not associated with depression (*p* = 0.438). Harmful alcohol users were more than five times likely to report conjoint tobacco and alcohol use (*p* = 0.000), but women were less likely to report it (*p* = 0.000).

**Conclusion:**

Conjoint tobacco and alcohol use is common among patients with HIV infection. Although not associated with positive screen for depression, its relationship with harmful alcohol use reiterates the need for an integrated tobacco and alcohol use screening and treatment strategy in the HIV treatment programme in primary care.

**Contribution:**

To the authors best knowledge, this study is the first published study that explored the relationship between conjoint tobacco and alcohol use, and depression among HIV-positive patients in the South African primary care settings.

## Introduction

Tobacco use and alcohol misuse are established independent risk factors for adverse health outcomes, and their combined or conjoint use may increase the risks of adverse health outcomes exponentially.^1^ This has huge clinical, financial and quality-of-life implications for patients and the healthcare systems.^2^ In the context of chronic diseases like human immunodeficiency virus (HIV), the need to adjust to physical symptoms such as pain, and psychosocial challenges such as stigma and acceptance of diagnosis, may promote substance use and misuse, and increase the risks of mental health disorders such as depression.^3,4,5^

South Africa has the largest HIV infection burdens in the world, making it a public health priority.^6,7^ Human immunodeficiency virus diagnosis has also been reported to be associated with social stigma, anxiety and depression.^3,4,5^ Depression as a comorbidity in the setting of HIV-related diseases could be challenging for the patient, often stretching the patient’s capacity to cope. This may in turn predispose the patient to unhealthy coping behaviours such as tobacco use and alcohol misuse. Each of these behaviours increases the risks of adverse health outcomes, and when used together (conjoint tobacco and alcohol use), these risks increase exponentially.^8^ In a study done among tuberculosis patients in a primary health care (PHC) facility in South Africa, the prevalence of conjoint tobacco and alcohol use was estimated at 10.1%.^9^ However, some of the participants in the study were HIV-negative or their HIV status were not known.

In South Africa, the predominant form of tobacco use is cigarette smoking^10,11^; other forms include smokeless tobacco (SLT) (snuff), waterpipe, e-cigarettes and cigars. In the recent Global Adult Tobacco Survey in South Africa published in 2022, the prevalence of tobacco use was 29.4%.^12^ According to the World Health Organization (WHO), cigarette smoking is the leading preventable risk factor for premature mortality. Cigarette smokers are likely to die younger than nonsmokers^13,14,15^ due to various adverse health effects such as cancers, respiratory and atherosclerotic cardiovascular diseases. Human immunodeficiency virus-positive patients who smoke tobacco are also more predisposed to tobacco attributable diseases.^16^

Alcohol is also a commonly used substance and its consumption is increasing in developing countries, including South Africa.^17^ In a national survey in South Africa, the prevalence of alcohol use was estimated at 41.5% (men) and 17.1% (women), respectively, with 9% of users reported to have problematic or harmful alcohol use.^18^ Harmful alcohol use constitutes huge health and economic burden worldwide with increased risks for cardiac problems, liver diseases, anxiety and depression. It was previously reported that there were health benefits from a ‘low or moderate’ level of alcohol consumption. These health benefits were thought to arise from the effects of alcohol on increasing cardioprotective high-density lipoproteins (HDL-C) and its effects on certain inflammatory markers.^8^ However, some subsequent studies have disputed these health benefits.^19,20^

Tobacco and alcohol use have substantial health and economic costs. In the United States, cigarette smoking causes 480 000 preventable deaths yearly^21^ and excessive alcohol drinking costs nearly $250 billion in 2010.^22^ According to the National Institute for Health and Care Excellence (NICE) in the United Kingdom, alcohol misuse costs the National Health Service (NHS), £3.5 billion (pound sterling) per year and resulted in 1.2 million hospital admissions and 15 000 deaths between 2010 and 2011.^23^ A study in South Africa reported that in the year 2016, an estimated 25 708 deaths were due to cigarette smoking, accounting for a healthcare cost of R14.48 billion.^24^ In a similar manner, the tangible financial cost of harmful alcohol use was estimated at R37.9 billion in 2009.^25^

As already alluded to, the physical demands and psychosocial dynamics of living with HIV, and the need to adjust, may increase not only the risk of depression but also that of substance use disorders, including conjoint tobacco and alcohol use. Despite this, no study in South Africa has explored the relationship between conjoint tobacco and alcohol use and depressive disorders among HIV-positive patients, particularly so in PHC, where most of these patients are cared for. The aim of this study was to determine the prevalence of conjoint tobacco and alcohol use and its association with screening positive for depression among HIV-positive patients in Sedibeng District. It is envisaged that the findings of this study may inform strategies aimed at addressing substance use and misuse and depressive disorders in the HIV treatment programme in South African PHC and similar settings.

## Methods

### Study design and setting

This was a cross-sectional study conducted during 2020–2021 at Levai Mbatha and Johan Heyns Community Health Centres (CHCs) located in Evaton township and Vanderbijlpark, respectively. Both CHCs render 24-h services, 7 days a week, and act as referral centre for several clinics. Levai Mbatha CHC serves a catchment population of about 90 724, mainly black patients.^26^ Johan Heyns serves a mixed racial population of 74 045.^26^ Both CHCs provide comprehensive PHC services including health promotion and prevention, curative care, maternity and child healthcare, rehabilitation and mental health services. A non-government organisation offers support to both CHCs on substance abuse prevention and HIV and/or acquired immunodeficiency syndrome (AIDS).

### Sample size and sampling

According to the TIER.NET information system, there were 4204 and 2105 adult HIV-positive patients receiving care at Levai Mbatha and Johan Heyns, respectively, during the period of this study. Using EPI INFO^TM^ Version 7.2.2.6 and assuming a 95% confidence interval, an expected frequency of 50% and a margin of error of 5%, the required sample size was estimated as 362. The final sample size was increased to 404 to compensate for any potential incomplete and missing data. This was allocated pro rata as 269 and 135 participants between Levai Mbatha and Johan Heyns CHCs, respectively.

All patients who were 18 years and older, and who were attending the HIV clinic at Levai Mbatha and Johan Heyns CHCs, were eligible to participate. To be included in the study, the patient must have given written consent. A participant information sheet was given to all consecutive potential participants, and the nature of the study was further explained to them by the principal investigator and trained research assistants. Thereafter, written consent was obtained from those who agreed to participate. Sampling enlisted consecutive patients until the sample size was completed.

### Measurement tools

A questionnaire composed of two parts was used to collect data. The first part collected sociodemographic information of participants, and any documented medical condition, such as diabetes, hypertension, chronic obstructive pulmonary disease, among others, was extracted from the participant’s file. The second part adapted three tools – Patient Health Questionnaire (PHQ-2 and PHQ-9), the Alcohol Use Disorder Identification Test (Consumption) AUDIT-C, and the Heaviness of Smoking Index (HSI):

- The PHQ-2 and PHQ-9 have been validated as a screening tool for depression in several studies in primary care settings both in South Africa and other countries.^27,28,29,30^ The PHQ-2 is a short version of PHQ-9 and consists of the first two questions of the PHQ-9 used for screening for depressive symptoms in the last 2 weeks. A zero score in the PHQ-2 excludes depression. A score of 3 or more screens positive for depression.^30,31,32^ The remaining seven questions of the PHQ-9 are thereafter administered to those who screen positive for depression, to categorise their depression (mild or severe) for appropriate management. The frequency of these symptoms over the days in the last 2 weeks are scored from: 0 = Not at all, 1 = Several days, 2 = More than half the days and 3 = Nearly every day. The maximum score is 27.
- The AUDIT-C tool was used to assess alcohol consumption among participants. This tool has been validated in studies, including those in primary care settings.^33,34^ The AUDIT-C tool is suitable for quick screening for harmful or hazardous alcohol use. A score of 5 or more indicates a positive screen.^35^ Its three-item score makes it less cumbersome yet an effective tool for screening for harmful alcohol consumption. The questions are asked on the typical frequency and quantity of alcohol consumption during the past year. Each question on the three items has five alphabetical answer choices from 0 to 4: (a = 0, b = 1, c = 2, d = 3 and e = 4), which gives a maximum total score of 12. One standard alcohol drink per unit is 10 g – 12 g of ethanol, equivalent to 340 mL – 355 mL of beer (5% by volume), 150 mL of wine (12% by volume) or 44 mL of spirits 40% by volume (80 proof).
- Tobacco use was assessed by self-report on the form of tobacco used, the quantity, the frequency of use and the duration. The HSI tool was used to assess for the presence or absence of nicotine dependence. The HSI is a short version of Fagerstrom Test for Nicotine Dependence (FTND), and its validity as a measurement tool for nicotine dependence among cigarette smokers has been shown in studies.^36^ Although a study found a positive correlation of FTND on SLT users,^37^ it has not been validated as a tool for assessing nicotine dependence for SLT use.

### Data collection process

The principal investigator liaised with the Health Promoters and Queue Marshals in each CHC, who daily informed patients about the study during the health talk. A trained research assistant who was fluent in the local languages (Zulu, Sotho, English and Afrikaans) assisted with interpretation during data collection. The research assistant approached the patients individually while they waited in the queue and engaged them on the nature and purpose of the study. Those who indicated interest were taken to the researcher in a private room nearby, where consent was obtained. Thereafter, the questionnaire was administered by the principal investigator. The trained research assistant acted as an interpreter where a participant did not understand English language. Information on medical conditions additional to HIV, was extracted from the participant’s medical record. A unique number code was assigned to each participant and a sticker was placed on the file to prevent resampling. The researcher then thanked the participants and returned them to their spots on the queue or the next top position.

### Analysis

All data collected in the paper-based questionnaires were entered into EPI Info^TM^ statistical software version 7.2.2.6 for analysis with the assistance of a biostatistician. Descriptive statistics were used to analyse the distribution of participants’ sociodemographic and clinical characteristics, and their tobacco and alcohol use patterns. Participants were divided into those that screened positive and negative for depression. Participants who reported alcohol use either conjointly with tobacco or alone were scored and categorised as harmful alcohol users or not. The relationship between conjoint tobacco and alcohol use and depression was explored with bivariate and logistic regression analyses. Statistical significance was set at *p* < 0.05.

### Ethical considerations

Ethics approval was obtained from Human Research Ethics Committee (HREC medical) of the University of the Witwatersrand (clearance number: M1911116). Permission was also obtained from the management of the Sedibeng district health services and facility managers of the study sites. To ensure anonymity, each participant was given a unique number code and there were no personal identifiers. A separate sheet that linked the code to the participant’s file number was kept securely and only accessible to the researcher.

## Results

A total of 410 eligible participants were approached but only 404 consented to participate. Six declined on account of pressure of time.

The sociodemographic and clinical characteristics of participants are presented in Table 1, which shows that the mean age was 43.2 years, with most participants completing secondary education (62.9%), being black people (99.0%), female (65.8%) and unemployed (53.6%). Most participants had been on antiretroviral therapy (ART) for more than 1 year (97.8%) and only 15.1% had comorbid conditions, of which hypertension was the most common (77.1%).

**TABLE 1:** Participants’ sociodemographic and clinical characteristics.

Variable	*n*	Percentage (%)	Mean ± s.d.	Range (years)
**Age**	-	-	43.2 ±11.6	19–77
**Age groups (years) (*N* = 404)**				
19–34	98	24.2	-	-
35–50	192	47.5	-	-
51–66	109	26.9	-	-
67 >	5	1.4	-	-
**Gender (*n* = 404)**				
Female	266	65.8	-	-
Male	138	34.2	-	-
**Population group (*n* = 404)**				
Black people	400	99.0	-	-
Mixed race people	1	0.3	-	-
White people	3	0.7	-	-
**Education level (*n* = 402)**				
None	16	3.9	-	-
Primary	80	19.9	-	-
Secondary/matric	253	62.9	-	-
Tertiary	53	13.1	-	-
**Marital status (*n* = 401)**				
Divorced/separated	51	12.8	-	-
Married/cohabit	124	30.9	-	-
Single	197	49.1	-	-
Widowed	29	7.2	-	-
**Employment status (*n* = 403)**				
Employed	162	40.2	-	-
Self-employed	25	6.2	-	-
Unemployed	216	53.6	-	-
**ART duration in years (*n* = 402)**				
< 1	9	2.2	-	-
1–10	319	79.4	-	-
11 >	74	18.4	-	-
**Presence of other medical conditions (*n* = 404)**				
Yes	61	15.1	-	-
No	343	84.9	-	-
**Type of other medical condition (*n* = 61)**				
Asthma	1	1.6	-	-
Depression	4	6.6	-	-
Diabetes	6	9.8	-	-
Hypertension	51	83.6	-	-
Epilepsy	1	1.6	-	-

Note: Type of other medical condition; total may exceed (*n*) since some patients had more than one medical condition.

The patterns of tobacco use are presented in Table 2. Current tobacco use was reported by 23.3% (94/404) with cigarette smoking being the most common form (73.7%, 70/94). Most cigarette smokers (75.5%; 53/70) had low nicotine dependence. Only 4.5% (18/404) of participants used SLT, and most were women (94.4%; 17/18) and dipped snuff 10 or fewer times a day.

**TABLE 2:** Tobacco and alcohol use pattern.

Category	Frequency	
	*n*	Percentage (%)
**All participants (*N* = 404)**		
• Alcohol only users	97	24.0
• Conjoint tobacco and alcohol users	79	19.6
• Tobacco only users	15	3.7
• Nonusers (no tobacco and no alcohol)	213	52.7
**Tobacco use**	94	23.3
• Cigarette smokers	70†(+1)	73.7
• Snuff users	18	18.9
• Water pipe users	7	7.4
**Heaviness of Smoking Index (HSI) among cigarette smokers (*n* = 70)**		
• High dependence	1	1.4
• Low dependence	53	75.7
• Moderate dependence	16	22.9
**Alcohol use**	176	43.6
• Hazardous and/or harmful drinking	65	36.9
• Nonhazardous drinking	111	63.1

† , (+1) = One participant who used waterpipe was also a cigarette smoker.

Current alcohol use was reported by 43.6% (176/404), with 36.9% (65/176) categorised as hazardous and/or harmful use.

The prevalence of conjoint tobacco and alcohol use was 19.6% (79/404).

In Table 3, an estimated 7.7% (31/404) of participants screened positive for depression, out of whom 71.0% (22/31) were categorised as having mild depression and most 83.3% (25/31) were previously undiagnosed and had not received any form of care or treatment.

**TABLE 3:** Results of screening for depression.

Variable	Frequency (*n*)	Percentage (%)
**PHQ-2 screen for depression Yes/No (*N* = 404)**		
Yes	31	7.7
No	373	92.3
**PHQ-9 depression classification (*N* = 31)**		
Mild – Minimal depression	22	71.0
Moderate – Moderately severe depression	8	25.8
Severe depression	1	3.2
**Gender distribution (*n* = 31)**		
Male	9	29.0
Female	22	71.0
**On treatment for depression (*n* = 30)**		
Yes	5	16.7
No	25	83.3

PHQ, Patient Health Questionnaire.

Bivariate analysis in Table 4 shows that there was no statistically significant association between depression and conjoint tobacco and alcohol use (odds ratio [OR] = 1.48, *p* = 0.438). However, those who were categorised as harmful alcohol users were more than five times likely to report conjoint tobacco and alcohol use (OR = 5.72; CI: 2.92 – 11.20; *p* = 0.000). In univariate logistic regression (Table 5), of all sociodemographic variables, being female (compared to male) was the only variable significantly associated with lower odds of conjoint tobacco and alcohol use (*p* = 0.000).

**TABLE 4:** Bivariate analysis: depression and conjoint tobacco and alcohol use.

Category	Conjoint tobacco and alcohol use		Total	Odds ratio	Confidence interval	*p*
	Yes	No				
**Depression**	**-**	**-**	**-**	1.484	0.544–4.043	0.438
Yes	9	8	17	-	-	-
No	69	91	160	-	-	-

**Total**	**78**	**99**	**177**	**-**	**-**	**-**

**TABLE 5:** Univariate logistic regression: sociodemographic variables and conjoint tobacco and alcohol use.

Variable	Odds ratio	95% CI	*p*
**Educational level**			
Ref. none	1	Reference	Reference
Primary	0.87	0.09–8.58	0.91
Secondary	0.82	0.09–7.68	0.86
Tertiary	0.90	0.08–9.70	0.93
**Employment status**			
Ref. employed	1	Reference	Reference
Self-employed	1.36	0.38–4.89	0.63
Unemployed	0.89	0.44–1.77	0.74
**Marital status**			
Ref. divorced	1	Reference	Reference
Married/cohabit	0.98	0.16–5.84	0.98
Separated	1.35	0.18–9.85	0.77
Single	1.24	0.21–7.29	0.81
Widowed	0.62	0.07–5.58	0.67
**Population group**			
Ref. black people	1	Reference	Reference
Mixed race	100180.98	0.00 – > 1.0E12	0.97
White people	0.00	0.00 – > 1.0E12	0.97
**Gender**			
Ref. male	1	Reference	Reference
Female	0.41	0.21–0.77	0.00

Ref., reference; CI, confidence interval.

## Discussion

This study found that conjoint tobacco and alcohol use is common among HIV-positive patients in primary care. Positive screen for depression is also found to be relatively common. While there was no statistically significant association between the two measures, participants who were harmful alcohol users were significantly more likely to report conjoint tobacco use.

The prevalence of conjoint tobacco and alcohol use in this study is higher than previously reported in South Africa (19.6% vs 10.1%),^9^ but is lower than a study in Thailand that reported a prevalence of 34.5%.^38^ The latter study was conducted among male hospital outpatients and could have had a higher prevalence because both lifestyle behaviours are more common among males than females.^9^ Although price and tax hikes are effective ways of curbing these lifestyles, a high prevalence of conjoint use in a setting of high unemployment suggests that the tax and rate hikes interventions in South Africa may not be strong enough deterrent. Additional policy interventions need to ensure that ‘sin taxes’ are aligned to wage increases and improve the enforcement of other tobacco control regulations. On the clinical front, the high prevalence underscores the urgent need for an integrated screening package that includes substance use and mental health within the HIV management programme.

The finding of no significant association between conjoint tobacco and alcohol use, and depression in this study, correlates with the report of a previous study that found that mental health problems were not among the factors associated with concurrent tobacco use and harmful alcohol use.^39^ While the results do not confirm the hypothesis that concurrent tobacco and alcohol use is associated with depression, it does highlight the prevalence of these risk factors and the gap in the health system for identifying and dealing with this. Previous associations between depression and tobacco and alcohol use have been mediated via poor health status,^40^ and may not apply now as access and coverage of ART have improved in South Africa, and consequently the health status of the HIV-postive population. However, conjoint tobacco and alcohol use and problematic alcohol use have been highly associated with nonadherence to treatment in another large South African study, with implications for poor HIV treatment outcomes.^41^ Considering the clinical and policy implications of self-medication with conjoint tobacco and alcohol use, more studies are required to clearly delineate the relationship between conjoint use of tobacco and alcohol and depression in South Africa especially as access, coverage and treatment outcomes improve.

Literature reports that the prevalence of tobacco use is higher among people with mental health problems than in the general population.^42^ However, this study found marginal differences in tobacco use and cigarette smoking prevalence between the study’s sample and the general South African population (23.3% vs 20.1% for tobacco use and 17.6% vs 17.3% for cigarette smoking).^10^ These minimal differences could be due to a virtually homogenous black population in this study that resulted in underrepresentation of other racial groups among whom smoking rates are much higher.^42,43^ It is perhaps important to note that a much higher prevalence of tobacco use (29.4%) was reported for the general population in the recent first South African Global Adults Tobacco Survey (GATS).^12^

Consistent with previous South African studies at population level,^44^ this study found low nicotine dependence among cigarette smokers, suggesting that HIV-positive patients, with or without depression, may be amenable to nonpharmacological smoking cessation interventions. Smokeless tobacco users in this study were mostly women, a finding consistent with the literature in South Africa.^45^ Although most of the snuff users reported dipping 10 or fewer times on a typical day and took their first dip after 60 minutes after waking up, nicotine is very addictive and may promote nicotine dependence.^14^ Smokeless tobacco also contains carcinogens that may increase the risk of many cancers, superimposed on an already elevated risk from HIV. Although there appears to be an increase in social acceptability of waterpipe tobacco use in South Africa, this study found a lower prevalence than reported in other South African studies.^46,47,48^ Summed together, irrespective of tobacco form, healthcare providers in South African PHC facilities need to offer tobacco cessation treatments to their patients who use tobacco, particularly brief motivational counselling.^44^

The prevalence of depression in this study was lower than previously reported in the general population (7.7% vs 9.7%).^49^ Landon Myer et al., however, reported a higher prevalence of depression of 14% among HIV-positive patients in a study conducted in Cape Town, South Africa.^50^ While this difference may be due to differences in measurement tool (PHQ-9 vs Centre for Epidemiological Studies Depression Scale [CES-D]), the underrepresentation of other racial groups in this study, among whom previous studies^49^ have reported higher prevalence of depression, could have resulted in the lower estimates. However, more proportions of females than males screened positive for depression in the study (71.0% vs 29.0%), consistent with the literature.^49^ Of note is that most of these participants had neither been diagnosed before nor received treatment underscoring the importance of targeting females with HIV for screening and diagnosis of depression, and once diagnosed, ensure that evidence-based management is instituted. Lastly, depression has been reported to be higher among individuals who are separated, divorced or widowed, compared to those who are married or living in a cohabit relationship^49^; however, this study found no statistically significant association between those who screened positive for depression and their marital status (*p* = 0.43).

Several potential limitations related to this study warrant discussion. Firstly, the consecutive sampling makes the study prone to sampling bias. Secondly, the tools used are self-reporting questionnaires and are prone to information and social desirability bias. Thirdly, because the study settings serve a predominantly black population group, other South African population groups were not proportionally represented, and this might have resulted in under- or overestimates, depending on the measures of interest. Caution should therefore be exercised in generalising the findings of this study to the general South African population. Breaking the bad news of HIV diagnosis could result in acute stress and adjustment disorders that may manifest with anxiety and depression symptoms. This may lead to overreporting of depressive symptoms by participants. However, such acute manifestation usually settles within a year of people knowing their HIV diagnosis, and in this study, almost all participants had known their HIV diagnosis and/or had been on ART for greater than a year before their participation in the study. Therefore, such influence was most unlikely. Regardless of the above potential limitations, this is one of the few studies that have explored the relationship between conjoint tobacco and alcohol use and depression among patients living with HIV in South African PHC, and the findings have implications for clinical practice and health policy for HIV, mental health and substance use disorder management.

## Conclusion

Conjoint tobacco and alcohol use is common among patients with HIV infection and significantly associated with hazardous/harmful alcohol consumption. These findings call for an integrated approach to strengthen the screening and treatment of tobacco and alcohol use disorders and depression among patients with HIV infection in South African PHC facilities and similar settings.
